# Oral versus patient-controlled intravenous administration of oxycodone for pain relief after cesarean section

**DOI:** 10.1007/s00404-019-05260-3

**Published:** 2019-08-17

**Authors:** Katja Mäkelä, Outi Palomäki, Satu Pokkinen, Arvi Yli-Hankala, Mika Helminen, Jukka Uotila

**Affiliations:** 1 Department of Obstetrics and Gynecology, Tampere University Hospital, PL 2000, 33521 Tampere, Finland; 2 Department of Anesthesia, Tampere University Hospital, PL 2000, 33521 Tampere, Finland; 3 Research, Development and Innovation Centre, Tampere University Hospital, PL 2000, 33521 Tampere, Finland; 4 Faculty of Social Sciences, Health Sciences, Tampere University, ARVO, 33014 Tampere, Finland; 5 Faculty of Medicine and Life Sciences, University of Tampere, Tampere, Finland

**Keywords:** Cesarean section, Oral analgesia, Oxycodone, Patient-controlled analgesia, Postoperative pain

## Abstract

**Purpose:**

The optimal postoperative analgesia after cesarean section (CS) remains to be determined. The primary objective of this study was to assess whether oral oxycodone provides the same or better pain control and satisfaction with pain relief as oxycodone given intravenously using a patient-controlled analgesia (PCA) infusion device. The secondary objectives were to compare the gastrointestinal symptoms and postsurgical recovery of the two groups.

**Methods:**

This prospective randomized trial was conducted at a University Hospital between February 2015 and June 2017. Altogether 270 CS patients were randomly assigned to receive postoperative oxycodone pain relief by IV PCA (*n* = 133) or orally (*n* = 137). Pain control and satisfaction with pain treatment were assessed by a numeric rating scale (NRS) at 2, 4, 8, and 24 h postoperatively.

**Results:**

No differences were found in NRS pain scores or satisfaction between the groups except at 24 h pain when coughing; there was a statistically significant difference favoring the IV PCA group (*p* = 0.006). In the IV PCA group, the patients experienced more nausea at 4 h (*p* = 0.001) and more vomiting at 8 h (*p* = 0.010). Otherwise, postoperative recovery was similar in both groups. The equianalgesic dose of oxycodone was significantly smaller in the oral group (*p* = 0.003).

**Conclusions:**

This study indicates that oral oxycodone provides pain control and satisfaction with pain relief equal to IV oxycodone PCA for postoperative analgesia after cesarean section. Satisfaction with pain treatment was high in both groups, and both methods were well tolerated. Early nausea was less common with oral medication.

## Introduction

Cesarean section (CS) is one of the most common surgical operations in the world [1]. In some countries, the CS rate reaches over 40% [2, 3]. In Finland, the CS rate has remained low at about 16.7% [4]. Pain relief after CS is crucial, and it affects both the mother and child. Poor pain relief may adversely affect recovery, mother–infant bonding, and breast-feeding; it may even lead to persistent postsurgical pain. Pain after CS was ranked ninth for pain severity among 179 surgical procedures in a study of 115,775 patients [5]. There are variable methods to manage postoperative pain, and the search for the ideal method is still ongoing. The use of opioids via different routes is the gold standard, although several other techniques have also been proposed [6].

After CS, patients usually need opioid analgesia for 1–2 days. Intrathecal, epidural, intravenous (IV), intramuscular (IM), subcutaneous, or oral routes and a continuous or intermittent manner of dosing can be used [7]. In addition to opioids, patients are given anti-inflammatory analgesics to promote better pain relief. The results of studies comparing different methods for pain relief after surgical interventions are controversial [8].

Neuraxial or IV morphine is considered the standard choice of opioid for post-CS pain [9]. IV oxycodone has gained increasing popularity of late due to its faster onset of action, fewer or less severe adverse events, and better effects for visceral pain [10]. Oxycodone has good oral bioavailability and a longer duration of action compared to morphine, thus making it a good choice for per oral pain relief [11]. A recent review of oral oxycodone for acute postoperative surgery showed that when administered as part of a multimodal analgesic regimen, it produces superior pain relief with fewer side effects and lower costs compared to epidural and IV analgesics [12].

In our institution, both oral and IV patient-controlled analgesia (PCA)-based pain relief are used. Our primary goal was to investigate by randomized controlled trial whether orally administered oxycodone provides pain control that is equal to or better than IV PCA oxycodone in CS patients. Our secondary objectives were to compare gastrointestinal symptoms and postsurgical recovery between the groups.

## Materials and methods

This prospective randomized study was conducted at the Department of Obstetrics and Gynaecology at Tampere University Hospital, Finland, between February 2015 and June 2017. During the study period, 1637 cesarean sections were conducted at the study hospital. The study protocol was approved by Pirkanmaa Hospital District’s ethics committee (Decision R 14090M, October 13, 2014).

**Fig. 1:**
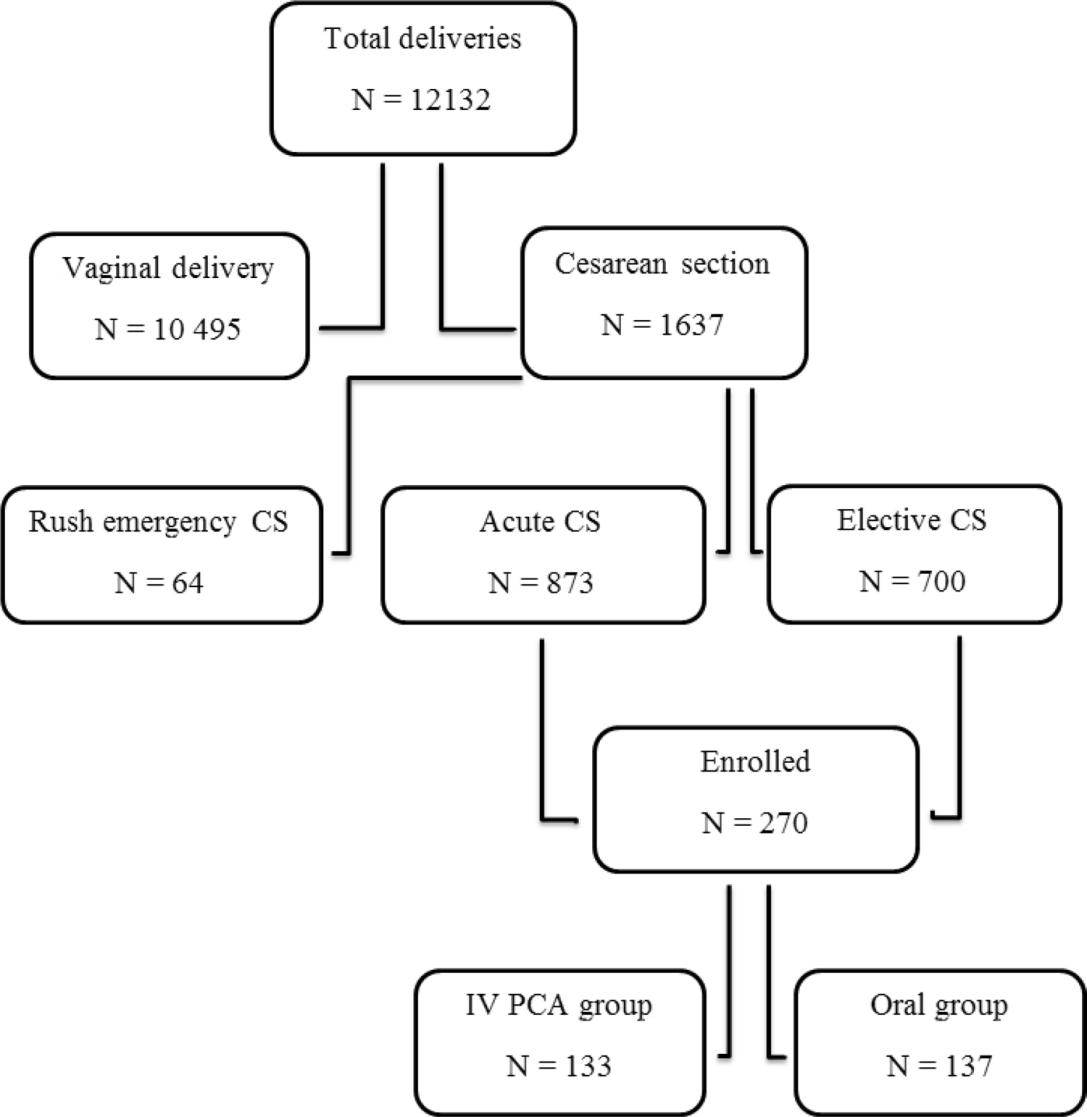
Distribution of patients during the study period

We sought to recruit women scheduled for elective or acute CS. Patients who underwent emergency CS or were unable to understand the Finnish language were excluded. Overall, 270 patients were randomized, 133 into the PCA group and 137 into the oral analgesia group. A flowchart of the recruitment process is shown in Fig. 1.

Patients were recruited to the study either in the outpatient clinic when the elective CS was arranged or in the delivery room when the decision for the acute CS was made. A written information letter and an oral explanation were given to the patients, and written consent was requested. A research nurse not participating in the treatment of the patients prepared envelopes including the information that assigned the patient to the IV PCA or oral group. A sealed opaque envelope was opened in the operating room by the operative nurse.

All patients were operated on under spinal anaesthesia. Spinal anaesthesia was performed using a 27-gauge BD™ Quincke spinal needle at the L3–4 level. The patients were given intrathecal 0.5% hyperbaric bupivacaine 11 mg and fentanyl 10 µg. Non-invasive arterial blood pressure was maintained above − 10% of the preoperative value using an intravenous crystalloid fluid infusion and boluses of intravenous phenylepinephrine 0.05 mg.

The patients had either a Pfannenstiel incision (263 patients) or a lower midline incision (3 patients in the IV PCA group and 4 in oral group).

Patients in both groups received extended-release oxycodone/naloxone 10/5 mg (OX/NAL) (oxycodone hydrochloride 10 mg + naloxone hydrochloride 5 mg), ibuprofen 600 mg, and paracetamol 1 g orally 1 h after surgery. Thereafter, an OX/NAL dose was given every 12 h, and ibuprofen and paracetamol were given every 8 h.

In the IV PCA group, the patients received an intravenous PCA device (CADD Legacy PCA, Smiths Medical MD, Inc., St. Paul, MN, USA) with oxycodone 1 mg/ml, using oxycodone bolus doses of 2 mg and a lockout time of 10 min. Patients were taught to use the pump in the operating theatre, and they were recommended to use it for at least 24 h.

**Fig. 2:**
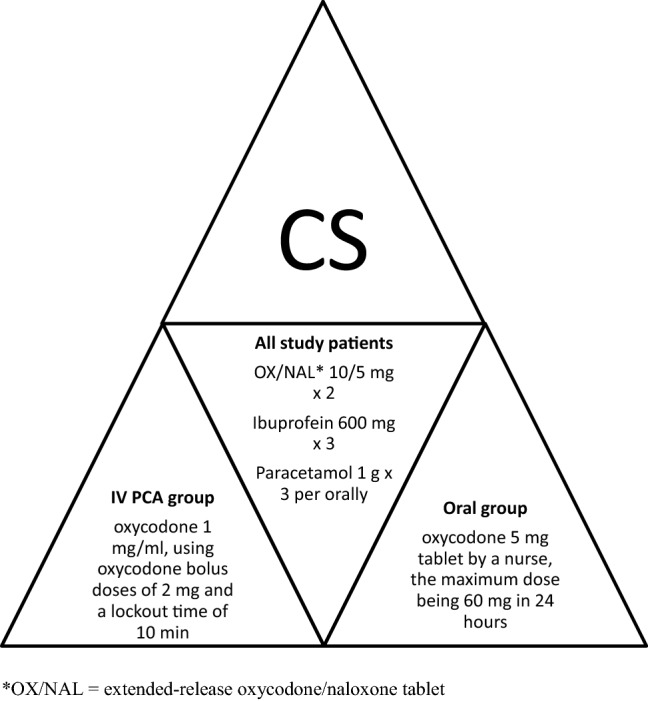
Postoperative medication protocol for the first 24 h after CS

In the oral analgesia group, patients were given an oxycodone 5 mg capsule upon request, the maximum dose being 60 mg in 24 h. The postoperative medication protocol is shown in Fig. 2.

Maternal pain scores and satisfaction with pain relief were asked and documented on the maternity ward. A numeric rating scale (NRS) was used. Pain and satisfaction were rated at 2, 4, 8, and 24 h after surgery. The pain scale ranged from 0 (= no pain) to 10 (= worst pain imaginable), and the satisfaction scale ranged from 0 (= completely dissatisfied) to 10 (= completely satisfied). Furthermore, the patient’s subjective perception about gastrointestinal symptoms—such as nausea, vomiting, and abdominal distension—was asked at 4, 8, and 24 h after surgery and categorized into two groups: patients with no symptoms and patients with symptoms. The time point for mobilization, the first meal, and defecation was recorded as well.

The equianalgesic dose ratio between intravenous and oral oxycodone has been reported to be 1:2 [13, 14]; this ratio was therefore used to calculate the equianalgesic dose of oral oxycodone for the two groups.

### Statistical analysis

Based on previous studies [15], the sample size calculations were made with the assumption that the standard deviations in pain scores would be 1.4, and a score difference between the groups of 0.5 would be regarded as clinically significant. With these assumptions, the sample size analysis indicated that 123 patients in each group would be sufficient.

Statistics were analysed using SPSS for Windows, version 22.0 (IBM Corp., Armonk, NY, USA, 2013). Variables were tested for normality using the Shapiro–Wilk and Kolmogorov–Smirnov tests. Differences in continuous variables were studied using Student's *t* test in cases of normality and by the Mann–Whitney *U* test in cases of skewed distribution. Categorical data are presented as percentages. They were compared using the Chi-square test or Fisher’s exact test, where appropriate. Intention to treat analyses was made by groups.

## Results

In total, 221 patients after elective CS and 49 patients after acute CS were enrolled in the study. The groups did not differ by characteristics regarding maternal background or the type of operation (Table 1).

**Table 1:** Demographic data and parameters concerning the mother, pregnancy, and operation

Characteristic	IV PCA group, *n* = 133		Oral group, *n* = 137	
	Median/*n*	Min–max/%	Median/*n*	Min–max/%
Age (years)	32	19–46	33	20–43
BMI (kg/m^2^)	23.6	16.2–49.5	24.6	17.5–52.6
Primipara	67	50.4	63	46.0
Gestational age at birth (days)	274	208–295	274	228–295
Full-term > 37 weeks	121	91.0	128	93.4
Prior CS	48	36.1	53	38.7
Elective CS	107	80.5	114	83.2
Operation time (min)	35	15–80	33	15–75
Bleeding (ml)	500	80–4600	450	100–3000

Values are expressed as the median or number of patients in the group and the parameter variables are expressed as the minimum to maximum or percentage in the group

Two patients in the IV PCA group needed general anesthesia during CS, one because of inadequate anesthesia and the other because of heavy bleeding during the operation. All other study patients were operated on under spinal anesthesia.

Five patients requested to have the IV PCA discontinued after a few hours’ use because of side effects like nausea. Respectively, six patients in the oral analgesia group switched to an IV PCA later on because of pain. Epidural analgesia was used for one patient in the IV PCA group, and two patients in the IV PCA group were given extra oxycodone for intolerable pain. The mean usage time of the IV PCA was 19 h postoperatively.

**Fig. 3:**
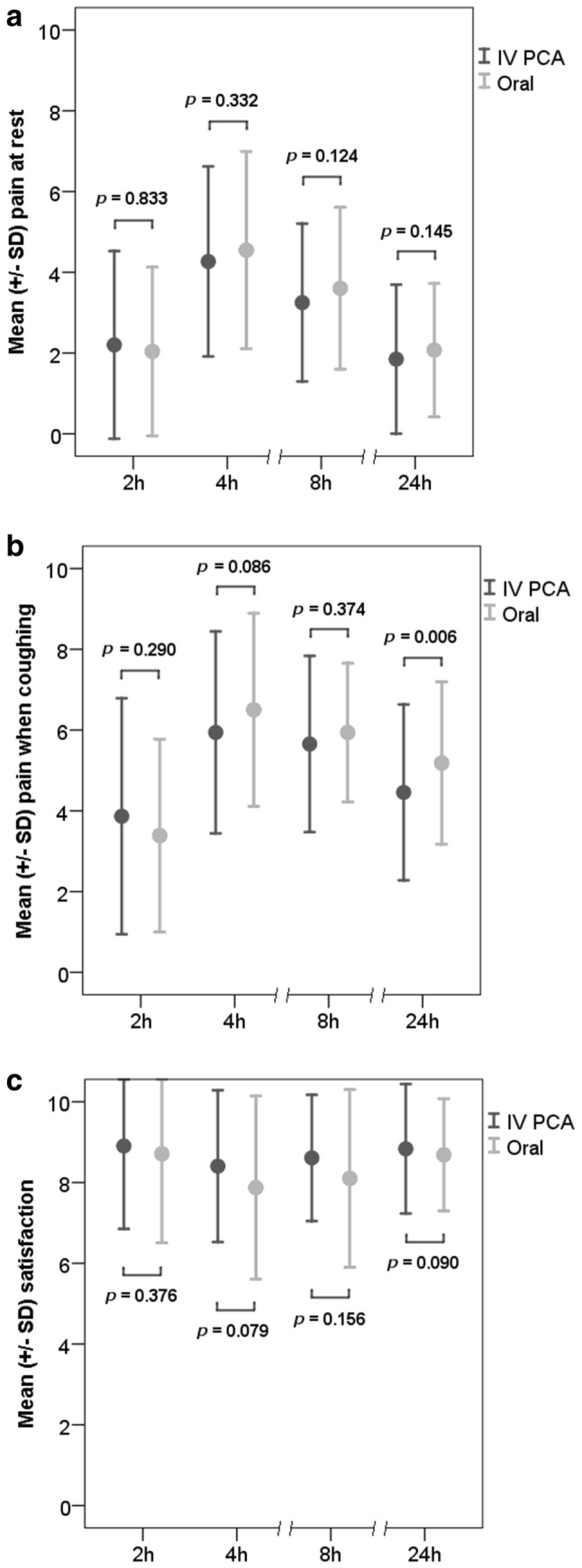
**a** Pain scores at rest at different time points after the operation (0 = no pain, 10 = the worst pain imaginable). **b** Pain scores when coughing at different time points after the operation (0 = no pain, 10 = the worst pain imaginable). **c** Satisfaction with pain relief at different time points after the operation (0 = completely dissatisfied, 10 = completely satisfied)

There were no differences in pain at rest or satisfaction between the groups at 2, 4, 8, and 24 h postoperatively, nor in pain when coughing at 2, 4, and 8 h postoperatively. At 24 h, the NRS for pain at coughing was higher in the oral group (*p* = 0.006; Fig. 3a–c).

To determine the most discontented patients in the groups, the proportions of women with severe pain (NRS ≥ 7) and dissatisfaction with pain treatment (NRS ≤ 3) were defined. At 24 h after CS, five patients suffered severe pain at rest in the IV PCA group, compared to none in the oral group (*p* = 0.026). No other differences were found between the groups either in the experience of severe pain or in dissatisfaction with pain treatment (Table 2). Overall satisfaction (NRS ≥ 7) for pain treatment was high: more than 77% were satisfied at every time point. There were no differences in severe pain or satisfaction between elective and acute CS patients.

**Table 2:** Severe pain (NRS ≥ 7) and dissatisfaction (NRS ≤ 3) at different time points

Characteristic	IV PCA group, *n* = 133		Oral group, *n* = 137		*p *value
Time	%	*n*	%	*n*	
Severe pain at rest					
2 h	8	10/119	3	4/124	0.083
4 h	21	26/123	24	30/126	0.614
8 h	8	9/120	7	8/121	0.788
24 h	5	5/106	0	0/111	0.026
Severe pain when coughing					
2 h	20	24/119	12	14/119	0.077
4 h	43	51/119	52	62/119	0.153
8 h	38	43/113	36	42/116	0.772
24 h	17	18/103	25	27/109	0.194
Dissatisfaction with pain treatment					
2 h	5	6/115	5	6/118	0.963
4 h	4	4/111	6	7/119	0.418
8 h	3	3/118	8	9/117	0.073
24 h	3	3/103	1	1/108	0.360

Patients in the IV PCA group had more nausea at 4 h (*p* = 0.001) and vomited more often at 8 h (*p* = 0.010). Otherwise, the groups did not differ in terms of gastrointestinal symptoms (Table 3). There was no difference in the recovery time of the groups (Table 4).

**Table 3:** Gastrointestinal symptoms at different time points after CS in the two groups

Characteristic	IV PCA group, *n* = 133		Oral group, *n* = 137		*p *value
Time	%	*n*	%	*n*	
Nausea 4 h	16	19/121	3	4/125	0.001
Nausea 8 h	9	11/121	5	6/120	0.215
Nausea 24 h	5	5/105	6	6/110	0.818
Vomiting 4 h	6	6/105	2	2/109	0.165
Vomiting 8 h	10	11/108	2	2/108	0.010
Vomiting 24 h	4	4/94	0	0/97	0.057
Abdominal distension 4 h	18	21/117	15	18/123	0.487
Abdominal distension 8 h	36	43/119	42	49/119	0.424
Abdominal distension 24 h	70	73/105	74	82/111	0.478

**Table 4:** Parameters concerning postoperative recovery expressed in hours

Characteristic	IV PCA group, *n* = 133		Oral group, *n* = 137		*p* value
	Mean	SD	Mean	SD	
First meal	7.8	5.5	7.0	4.6	0.336
Mobilization	17.4	7.4	17.8	6.7	0.567
Defecation	60.1	18.3	59.9	17.5	0.945

In the IV PCA group, the mean consumption of iv oxycodone was 58.2 mg (SD 23.5), while in the oral group, the mean counted equianalgesic dose was 48.3 mg (SD 11.8) during the first 24 h (*p* = 0.003).

## Discussion

In this prospective randomized study, we found that oral oxycodone was an equally effective and satisfactory means for postoperative analgesia after uncomplicated cesarean section as IV oxycodone administered by a patient-controlled device (PCA). However, nausea and vomiting were slightly more common in patients receiving IV PCA. Regarding postoperative pain scores and general satisfaction with pain relief, both methods were equally accepted by the patients. The results of our study are in line with those of Dieterich et al., who randomized 239 patients to receive either intravenous opioid piritramide (PCA) or oral oxycodone [16]. They used a visual pain scale (VAS) for pain assessment and found no differences between the groups. General satisfaction was also high, and Dieterich et al.’s results support the use of oral pain treatment after CS. Davis et al. also found that oral analgesia with oxycodone and acetaminophen after CS gave better pain relief with fewer side effects than an IV morphine PCA [17]. To our knowledge, no earlier studies comparing the per oral and IV PCA administration of oxycodone for post-cesarean pain and patient satisfaction exist.

Although alternative drugs, new combinations of existing drugs, and new applications of local anesthetics have been studied intensively, opioids are still required for post-cesarean analgesia [9]. Intravenous oxycodone is comparable to other IV opioids in terms of safety and efficacy [10], but even short-term opioid use by patients undergoing surgery can lead to chronic opioid use [18]. It is generally known that rapidly affecting drugs increase this risk, therefore IV administration might be unfavorable. Moreover, the cost of the IV PCA, including medicine, infusion liquid and PCA device, is about twice as high as the oral medication.

It is challenging to treat postoperative pain efficiently without side effects. Especially after CS, one must consider not only the pain relief and wellbeing of the mother, but also active mother–child bonding and breast-feeding. It is also crucial to take into account the high risk of thromboembolism after CS [19]. Since delayed standing can negatively influence these goals, early postoperative mobilization and immediate removal of the urinary catheter are recommended after CS [20, 21]. Thus, continuous epidural analgesia may not be favored, even if it might give excellent pain control. Intramuscular injection should be avoided, and transdermal opioids are appropriate only for persistent pain.

Self-administration with an IV PCA is the standard treatment in some hospitals, whereas oral administration is considered easier and practical in others. Compared to per oral administration, patients with an IV PCA can receive a drug therapy that is more individualized [22]. They receive immediate relief when needed, even for the slightest pain. However, unwanted side effects like nausea and drowsiness may delay recovery [17]. Per oral administration causes fewer side effects, but the pharmacological effect appears with a certain delay. Patients are also dependent on nurses, who are not always immediately available to deliver oral medication upon the patient’s request. Nevertheless, in this study, the patients in both groups were equally satisfied with the pain relief.

As part of our study protocol, all patients received oral ibuprofen and paracetamol regularly every eight hours. Combined with the opioid, their safety and opioid-sparing effect have been documented in many studies. In the IV PCA group, patients used more oxycodone compared to the patients in the oral group. Regardless, there were no major differences between the groups in terms of the NRS, postoperative recovery, or satisfaction. The protocol of this study included per oral postoperative extended-release oxycodone, which entailed constant oxycodone content for all patients. By combining this with a non-steroidal anti-inflammatory drug and paracetamol regularly, the need for short-acting extra oxycodone is considerably lower after surgery. This may be the reason why oxycodone administrated either by an IV PCA or orally ensured equal pain relief in our results. As is generally known, all patients usually benefit from participating in a clinical trial, regardless of the randomization.

When using an IV PCA, patients may have more nausea and drowsiness than with per oral analgesia [17], and this can negatively affect mobilization and baby care. Overall, nausea and vomiting after CS were rare events in our study compared to the study of Kim et al., where 127 patients had either an oxycodone or fentanyl IV PCA after a laparoscopic supracervical hysterectomy. Oxycodone was associated with superior analgesia, but those patients had a significantly higher incidence of side effects, such as postoperative nausea and vomiting, dizziness, and drowsiness [23]. In our study, over half the patients (*n* = 155/216) reported abdominal distension 24 h after surgery (no difference between study groups). Patients in the PCA group experienced more nausea at the 4-hour time point. Postoperative recovery was equal in both groups. The mean time of mobilization was 17 h, which is an exceptionally long time compared to our 6-h recommendation. Postoperatively, the patients were given meals depending on the schedule of the ward, i.e., the time of operation determined the time that the meal was offered. The mean delay was 7 h postoperatively. The Cochrane review in 2014 found early postoperative feeding after major gynecological surgery to be safe and to enable the faster recovery of bowel function, a shorter hospital stay, and higher satisfaction [24].

The strengths of this study include the large, randomized, non-selected material, and the comparison of two treatments that are normal in clinical use. A potential weakness of this study was the relatively low recruitment percentage of acute CS patients, which was 19.5% (26/133) in the IV PCA group and 16.8% (23/137) in the oral group. The difference between pain relief and postoperative recovery between the elective and acute CS could not be reliably estimated.

In conclusion, the results of this study show that oral oxycodone is an effective and satisfactory method for postoperative analgesia after cesarean section. IV oxycodone administered with the use of a PCA did not offer clinically significant benefits. Both methods were generally well tolerated, and pain relief was good with both methods. Early nausea was less common with oral medication. Therefore, our findings do not support the routine use of a IV oxycodone PCA in this patient group.

## Abbreviations

CS: Cesarean section
NRS: Numeric rating scale
PCA: Patient-controlled analgesia
NCA: Nurse-controlled analgesia
IV: Intravenous
IM: Intramuscular
VAS: Visual analogue scale
OX/NAL: Extended-release oxycodone/naloxone tablet
